# Newborn screening for Fabry disease in Japan: an additional 3-year report

**DOI:** 10.1016/j.ymgmr.2026.101327

**Published:** 2026-06-11

**Authors:** Takaaki Sawada, Jun Kido, Keishin Sugawara, Shinichiro Yoshida, Takahito Inoue, Shinichi Hirose, Kimitoshi Nakamura

**Affiliations:** a Center for Clinical Genetics, Kumamoto University Hospital, Kumamoto, Japan; b Department of Pediatrics, Faculty of Life Sciences, Kumamoto University, Kumamoto, Japan; c KM Biologics Co., Ltd., Kumamoto, Japan; d Center for Maternal, Fetal and Neonatal Medicine, Fukuoka University Hospital, Fukuoka, Japan; e General Medical Research Center, School of Medicine, Fukuoka University, Fukuoka, Japan

**Keywords:** α-Gal A, *GLA*, Fabry disease, Hypohidrosis, Newborn screening, Zebra bodies

## Abstract

Newborn screening (NBS) for Fabry disease (FD) is highly effective at detecting FD prior to symptom onset. This initiative is currently being implemented worldwide. We previously reported results for 599,711 newborns from the first large-scale NBS program for FD in Japan, from August 2006 to December 2018. In this study, we provide additional data from January 2019 to September 2022. A total of 782,591 newborns were screened, and 29 variants, including 18 pathogenic variants and 11 variants of uncertain significance (VUS), were detected in 77 newborns (57 males and 20 females). Thirty-five male and 14 female newborns with pathogenic variants in *GLA* were identified. Twenty-two male and six female newborns with VUS in *GLA* were also identified. At the most recent follow-up, 5 of the 35 hemizygous patients manifested symptoms or signs and were receiving enzyme replacement therapy. The estimated frequency of patients with FD, including individuals with pathogenic variants or VUS identified in this study, was 1 in 7730, whereas that of patients with pathogenic variants was 1:12,436. FD-related cardiac and renal tissue damage are present before the onset of FD symptoms, such as limb pain. These findings highlight the importance of early diagnosis using NBS and regular monitoring.

## Highlights

• The frequency of FD (pathogenic variants + VUS) in Japan was estimated to be 1:7730
• This study highlights the importance of early diagnosis using NBS
• We detected 29 *GLA* variants in 77 individuals

## 1 Introduction

Fabry disease (FD; OMIM 301500) is an inherited X-linked disorder caused by mutations in the *GLA* gene, which encodes the lysosomal enzyme α-galactosidase A (α-Gal A, EC 3.2.1.22). An α-Gal A defect leads to progressive accumulation of metabolites, such as globotriaosylceramide (Gb3) and globotriaosylsphingosine (lyso-Gb3) in lysosomes, which cause progressive dysfunction in systemic organs, including the skin, eyes, vessels, ears, lungs, kidneys, heart, and brain [1], [2], [3]. Male patients with very low α-Gal A activity often develop the classic phenotype. However, they may be asymptomatic until early childhood (mean age of onset, 9 years) [4], [5]. Various clinical manifestations, such as pain in the peripheral extremities, angiokeratoma, hypohidrosis, corneal opacity, cardiac disease, cerebrovascular disease, and kidney injury, develop with advancing age, and premature death may occur.

Male patients with FD and residual α-Gal A activity are likely to present with milder clinical manifestations with a later age of onset than in patients with classic Fabry. Their onset occurred later than in patients with the classic type. Females with heterozygous pathogenic variants may exhibit a broad spectrum of clinical manifestations, ranging from asymptomatic to as severe as those of classic patients, depending on random X-chromosomal inactivation [6], [7]. A total of 1285 variant have been registered in the public database (Fabry database. org, last updated on May 10, 2021, ver.3.5) [8], of which 725 have been reported as classic or severe-type mutations.

Several clinical expert opinions and guidelines have recommended regular monitoring and early evaluation of pediatric patients with Fabry disease, even before the onset of overt symptoms. These recommendations include periodic assessment of renal, cardiac, neurologic, ophthalmologic, and gastrointestinal manifestations, as well as consideration of early initiation of enzyme replacement therapy (ERT) in selected patients with classical Fabry disease [9], [10]. Therefore, newborn screening programs may provide an important opportunity for early diagnosis, longitudinal follow-up, and timely therapeutic intervention in affected individuals.

ERT is effective in slowing the progression of renal disease and cardiomyopathy and in preventing morbidity and mortality [11]. Several studies have suggested that initiating ERT before the onset of renal or cardiac failure yields optimal results [12], [13]. Three enzyme products (Fabrazyme®, Sanofi S.A., Paris, France, Replagal®, Takeda Pharmaceuticals Inc., Lexington, MA, USA, and Agalsidase beta BS®, JCR Pharmaceuticals Co., Ltd., Hyogo, Japan) also are approved for clinical use in Japan. Pharmacological chaperone therapy (Galafold®, Amicus Therapeutics K·K, Tokyo, Japan) can be orally administered, although the indication is limited to those aged >12 years with limited *GLA* gene variants [14]. Early treatment is critical to preserve organ function; however, many patients are often diagnosed at later stages or misdiagnosed owing to nonspecific clinical manifestations in the early stages of the disease [15].

Newborn screening (NBS) for FD aims to identify patients at risk for FD at an early stage before they present with manifestations. This approach is being implemented worldwide, improving patient quality of life through early detection and intervention [10]. In a previous study, we reported NBS in 599,711 newborns from the western region of Japan between August 2006 and December 2018 [16]. Herein, we present the results of NBS for an additional 182,880 newborns and report the outcomes of previously reported individuals identified via NBS harboring pathogenic *GLA* variants. Moreover, we discuss the genetic significance of some *GLA* variants and the importance of our NBS program for FD.

## 2 Materials and methods

### 2.1 Study population

The study population consisted of 182,880 newborns born between January 2019 and September 2022 in the Kumamoto and Fukuoka prefectures of Japan. The numbers of male and female newborns were 93,210 and 89,670, respectively. Dried blood spots (DBSs) from newborns were prepared at maternity clinics or obstetric departments using standard procedures at 4–6 days after birth. The DBSs were dried for at least 4 h at room temperature after blood spots were dropped onto filter paper (Toyo Roshi Kaisha, Ltd., Tokyo, Japan), and the dried DBSs were sent to KM Biologics Co., Ltd. by mail.

### 2.2 NBS for FD

**Fig. 1:**
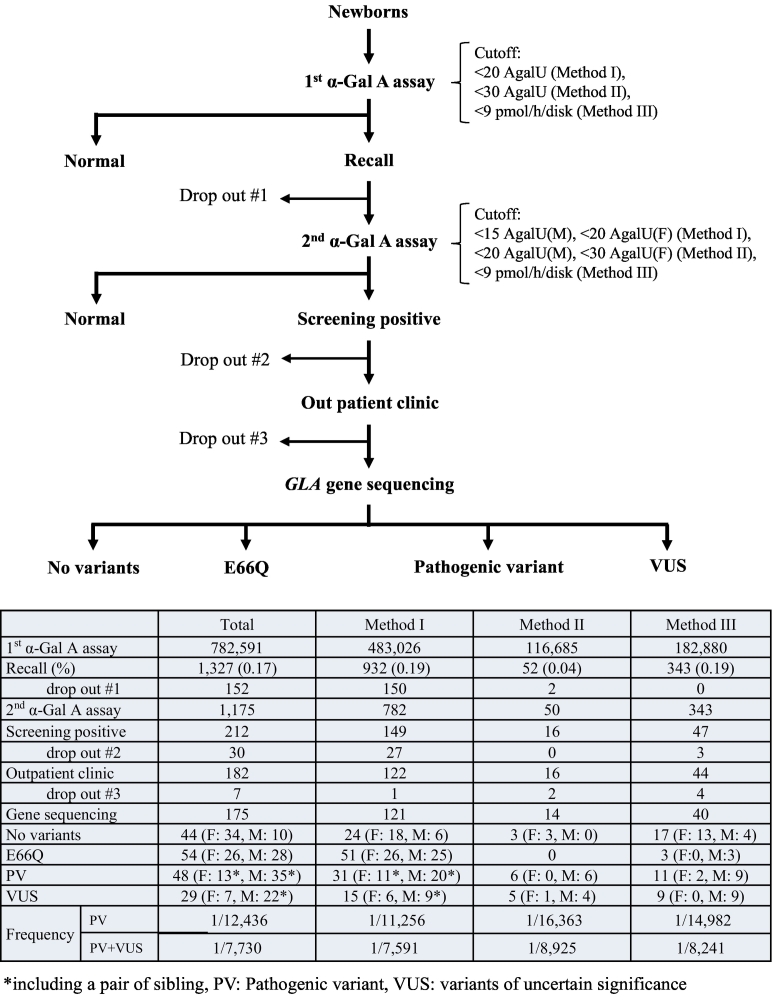
Flowchart and results of newborn screening for Fabry disease.

**Fig. 2:**
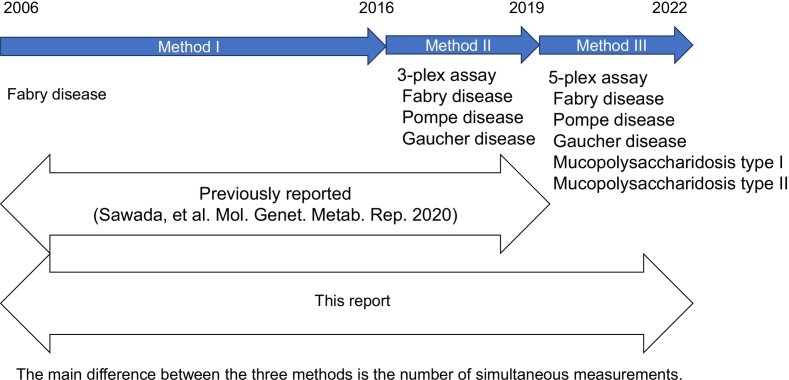
Changes in methods and the position of this report.

The NBS using α-Gal A activity assays in the DBSs were performed using three methods (Fig. 1, Fig. 2). From August 2006 to October 2016, α-Gal A assays were performed using Method I. From November 2016 to December 2018, α-Gal A activity was analyzed using Method II. Since January 2019, α-Gal A activity has been analyzed using Method III.

These NBS methods were modified to optimize the extraction of multiple enzymes from DBSs (Fig. 2).

### 2.3 α-Gal A assay

The procedures for Methods I and II for α-Gal A assays using DBSs were described in our previous report [16].

#### 2.3.1 Method I

Briefly, a single 3.2-mm diameter disk punched from DBSs was incubated in a well of a 96-well clear microwell plate (Corning, NY, USA) with 40 μL Mcilvaine buffer (100 mM citrate, 200 mM NaH_2_PO_4_, 36.8:63.2, pH 6.0) and processed for extraction at room temperature for 2 h. Aliquots of 30 μL blood extract were transferred to fresh 96-well microwell plates. An aliquot of 100 μL of the reaction mixture (3.5 mM 4-methylumbelliferyl-α-d-galactopyranoside (4MU-αGal), 100 mM citrate, 200 mM K_2_HPO_4_, 100 mM *N*-acetyl-*d*-galactosamine) was added to each well of the microwell plates and incubated at 37 °C for 24 h. The reaction was terminated with 150 μL termination solution (300 mM glycine/NaOH, pH 10.6) immediately after the reaction. The fluorescence intensity of 4-methylumbelliferones in the wells was measured using a fluorescence plate reader (BIO-TEK, Winooski, VT, USA) at 450 nm. One unit (1 AgalU) of enzymatic activity was equal to 0.34 pmol of 4MU-αGal cleaved/h per disc.

#### 2.3.2 Method II

Method II was developed for high-throughput, multi-assay testing in collaboration with KM Biologics Co., Ltd., and implemented from November 2016 to December 2018. Briefly, a single 3.2-mm diameter disk punched from DBSs was incubated in a well of a 96-well clear microwell plate (AS ONE Corporation, Osaka, Japan) with 100 μL of 25 mM citrate/potassium phosphate buffer (pH 6.0) containing 5 mM MgCl_2_, 0.5 mM dithiothreitol, 0.05% NaN_3_, and 0.1% Triton X-100 for 1 h at room temperature with gentle mixing. A 20-μL aliquot of the extract was then added to 40 μL of the reaction mixture (3.0 mM 4MU-αGal, 100 mM *N*-acetyl-*d*-galactosamine in 100 mM citrate/200 mM potassium phosphate buffer, pH 4.4) in a 96-well black microwell plate (Thermo Fisher Scientific Inc., MA, USA). The reaction mixture was incubated at 38 °C for 3 h, and the reaction was stopped by adding 200 μL of 300 mM glycine/NaOH buffer (pH 10.6) containing 10 mM ethylenediaminetetraacetic acid to measure fluorescence intensity. This residual extract could be used for the assay of acid α-glucosidase (Pompe disease) and glucocerebrosidase (Gaucher disease) activities.

#### 2.3.3 Method III

Method III was performed using the Enzaplate Lysosomal Storage Diseases (LSD) assay kit (Siemens Healthcare Diagnostics K.K., Tokyo, Japan) and was implemented in January 2019. The kit was developed by Daiichi Kishimoto Clinical Laboratories (Sapporo, Japan) and Sapporo Immuno Diagnostic Laboratory Co., Ltd. using intellectual property JP6989872 under the license of KM Biologics Co., Ltd. Using this kit, five LSD, Fabry, Pompe, Gaucher, mucopolysaccharidoses (MPS) 1, and MPS 2, were assayed using a single 3.2 mm diameter disk punched from DBS.

Briefly, a single 3.2 mm diameter disks punched from DBS was incubated in a 96-well clear microwell-plate with 200 μL of extraction solution (included in the kit). In a 96-well black microwell plate, a 20 μL aliquot of the extract was added to 40 μL of substrate solution (included in the kit) and incubated at 38 °C for 3 h, after which 200 μL of reaction stop solution (included in the kit) was added. The fluorescence intensity was analyzed at excitation and emission wavelengths of 370 and 465 nm, respectively. The molar product quantities in the assay wells were calculated from the standard curve using linear regression. Enzymatic activity was expressed as picomoles of 4-MU released per hour per disk (pmol/h/disk).

### 2.4 The flow-chart of NBS for FD

Newborns with α-Gal A activity under the cutoff level in the DBSs (< 20 [AgalU] for Method I, < 30 [AgalU] for Method II, < 9 [pmol/h/disk] for Method III) were recalled, and their DBSs were prepared again. One AgalU sample is equal to 0.34 pmol/h/disk. In the second step, newborns with α-Gal A activity under the cutoff level (Method I: < 15 [AgalU] for males, < 20 [AgalU] for females; Method II: < 20 [AgalU] for males, < 30 [AgalU] for females; Method III: <9 [pmol/h/disk] for both males and females) were called to the hospital for clinical examinations (Fig. 1). Newborns were examined using physical and biochemical evaluation including complete blood counts, aspartate aminotransferase, alanine aminotransferase, blood urea nitrogen, creatinine, sodium, potassium, and chloride to confirm the presence of FD symptoms. *GLA* gene sequencing was performed on newborns whose parents provided informed consent.

### 2.5 Sequencing of the GLA gene

#### 2.5.1 Sanger method

Genomic DNA was extracted from total blood using a Gentra Puregene Blood Kit (Qiagen, Hilden, Germany) or equivalent product and stored at −80 °C until use. All seven exons and flanking intronic sequences of the *GLA* gene were amplified via polymerase chain reaction (PCR) [17], [18]. The intron 4 sequence was amplified to detect the c.640-801G > A variant [19]. PCR products were sequenced on an ABI3500xl autosequencer (Applied Biosystems, Foster City, CA, USA) and analyzed using Sequencher 5.0 (Gene Codes Corporation, Ann Arbor, MI, USA).

#### 2.5.2 Next-generation sequencing (NGS)

Sequencing of the *GLA* gene using NGS was conducted as described in our previous reports [16], [20]. Briefly, the 13.3-kbp region, including the *GLA* gene, was amplified via long-range PCR. Library preparation and sequencing were performed using the Nextera XT Kit (Illumina, San Diego, CA, USA) and a MiSeq sequencer (Illumina). After sequencing runs, the data were aligned to the human reference genome sequence (NC_000023.11) using MiSeq Reporter software (Illumina).

Sequence data analysis, mapping, and variant calling were streamlined using the MiSeq Reporter v2 (Illumina). The sequencing reads were visualized using IGV_2.8.0 (Broad Institute). Variants detected in the *GLA* gene by NGS were resequenced using the Sanger method.

### 2.6 Mutation analysis of the variants

The mRNA reference sequence RefSeq NM_000169.3 was used for this study; the “A” nucleotide of the ATG codon at nucleotide position 111 of the RefSeq sequence constituted +1 numbering of the cDNA sequence. The ATG codon also represented +1 for the amino acid numbering as set forth by the α-Gal A preprotein sequence NP_000160.1. The variant nomenclature followed the guidelines established by the Human Genome Variation Society (http://varnomen.hgvs.org/). Public databases, including Fabry-database.org [8] (http://fabry-database.org/, updated May 10, 2021) and ClinVar [21] (http://www.ncbi.nlm.nih.gov/clinvar), were used to classify each variant. The software PolyPhen-2 [22] (http://genetics.bwh.harvard.edu/pph2) was used for missense variants to predict the potential impact of alterations in amino acids on the function of α-Gal A.

### 2.7 Statistical analyses

The α-Gal A activities obtained from Methods I, II, and III were compared between male and female newborns using *t*-tests. Statistical analyses were performed using IBM SPSS software version 25 (IBM Corporation, Armonk, NY, USA). Results with *P* values of <0.05 were considered statistically significant.

### 2.8 Follow-up survey

We conducted a follow-up survey to assess the status of patients diagnosed with FD and those with a pathogenic variant or VUS in *GLA*. The current status of the patients or individuals attending Kumamoto University Hospital was confirmed from their medical records. Other patients or clinicians were interviewed via email regarding the symptoms and signs of FD, and information on interventions, including ERT and chaperone therapy, was also collected via email.

### 2.9 Ethics

This study was approved by the Ethics Committee of Kumamoto University (approval no. 1537). Written informed consent was obtained from the parents or legal guardians of newborns.

## 3 Results

### 3.1 NBS for FD

**Fig. 3:**
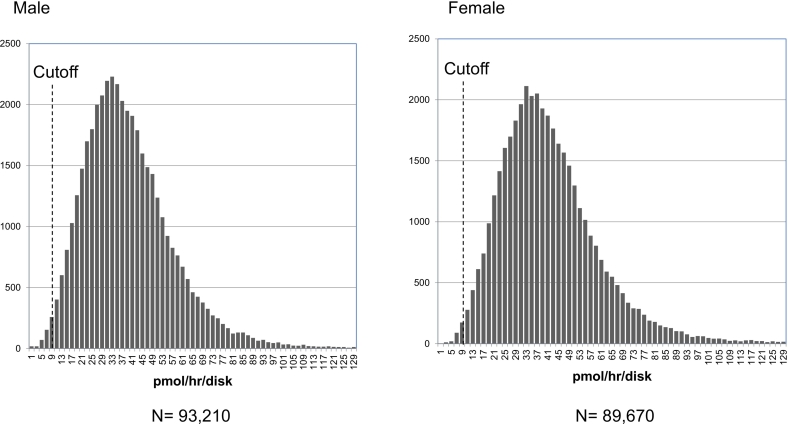
Histograms of α-Gal A activity in newborns (Method III). Male (*N* = 93,210) and female (*N* = 89,670) distributions are shown. Dashed line indicates the cutoff level (9 pmol/h/disk).

A flowchart and results of the NBS program for FD are shown in Fig. 1, Fig. 2, respectively. In total, 782,591 newborns (483,026 in Method I, 116,685 in Method II, and 182,880 in Method III) were screened. The mean and median α-Gal A activities obtained using Methods I and II have been previously described [16]. The mean and median activities obtained using Method III were 39.87 ± 19.93 (pmol/h/disk) and 36.90 (pmol/h/disk; interquartile range [IQR]: 27.10–49.00), respectively, in male newborns and 42.03 ± 20.11 (pmol/h/disk) and 39.00 (pmol/h/disk; IQR: 28.90–51.10), respectively, in female newborns (Fig. 3). The α-Gal A activities of female newborns were slightly higher than those of male newborns in Methods I–III (all *p* < 0.001).

Next, a total of 1327 (0.17%) newborns in Methods I–III were recalled for the second α-Gal A assay, and 182 newborns with low α-Gal A activities under the cutoff at the second α-Gal A assay were examined at outpatient clinics (Fig. 1). These newborns were evaluated by physical and laboratory examinations, and *GLA* gene sequencing was performed in 175. Forty-four newborns presented with no variants of the *GLA* gene, 54 newborns presented with a functional polymorphic allele, p.E66Q [23], [24], and 77 newborns, including 3 siblings, presented with pathogenic variants or VUS. The estimated frequency of patients with FD, including individuals with pathogenic variants or VUS identified in this study, was 1 in 7730 (0.013%), whereas that of pathogenic variants was 1 per 12,436 births (0.008%).

### 3.2 Variants detected in newborns

The *GLA* gene is highly polymorphic; hence, many novel variants are likely to be discovered. A total of 1061 and 1285 variants are registered in ClinVar and Fabry-database.org, respectively. Eleven variants including pathogenic and VUS were identified among 20 newborns detected in the Methods III (Fig. 1 and Table 1). Consistent with previous findings, 29 variants (pathogenic or VUS) were identified in 77 newborns (Table 2). Twenty-five of the 29 variants were missense mutations, 3 were in-frame deletions, and 1 was an intronic mutation. The phenotypes of these 29 variants were assigned using the Fabry database.org. Seven of these 29 variants, i.e., c.2 T > A p.M1K [16], c.124 A > G p.M42V [25], c.128G > A p.G43D [26], c.595G > A p.V199M [26], c.719delA p.K240Rfs*29 [27], c.761_763delTTG p.V254del [28], and c.1072_1074delGAG p.E358del [29], were assigned as classic-type mutation. Ten variants, i.e., c.290C > T p.A97V [28], c.335G > A p.R112H [24], [30], c.436C > T p.P146S [31], c.628C > T p.P210S [32], c.640-801G > A [19], c.644 A > G p.N215S [33], c.685 T > G p.F229V [34], c.888G > A p.M296I [35], c.899 T > C p.L300P [36], and c.1171 A > G p.K391E [24], [37] were assigned as mild/later-onset type mutation. Three variants, c.431G > A p.G144D, c.687 T > G p.F229L, and c.1208 T > C p.L403S, were reported by Li et al. in a Chinese FD patient [38], Kawano et al. in a male patient with cardiac FD diagnosed at 60-years old [39], and Shimotori et al. in a Japanese FD patient [25]. We classified 20 of the 29 variants as pathogenic and the remaining 9 as VUS according to public databases, including Fabry-database.org [8] and ClinVar [21] (Table 2).

**Table 1:**
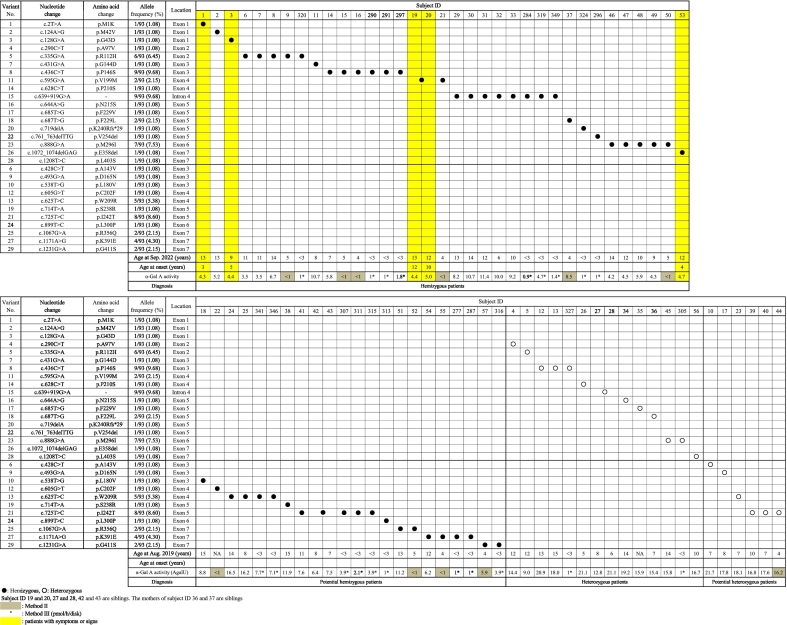
The variants detected in newborn screening for Fabry disease.

**Table 2:** Variants detected in the newborn screening program for Fabry disease.

No.	Nucleic acid	Amino acid	Location	ClinVar	Fabry-database.org*	Polyphen-2 (Score)	Amenability**	Allele frequency***	References
**1**	**c.2 T > A**	**p.M1K**	**Exon 1**	**NR**	**Classic**	**Benign (0.080)**	**−**	**NR**	**Sawada (2020) [15]**
2	c.124 A > G	p.M42V	Exon 1	Pathogenic	Classic	Probably damaging (1.000)	+	NR	Shimotori (2008) [25]
**3**	**c.128G > A**	**p.G43D**	**Exon 1**	**NR**	**Classic**	**Probably damaging (1.000)**	**−**	**NR**	**Shabbeer (2002) [26]**
4	c.290C > T	p.A97V	Exon 2	Pathogenic	Mild	Possibly damaging (0.882)	+	NR	Eng (1997) [29]
5	c.335G > A	p.R112H	Exon 2	Pathogenic	Mild	Probably damaging (1.000)	+	NA	Eng (1994) [30]
6	c.428C > T	p.A143V	Exon 3	NR	VUS?	Probably damaging (1.000)	+	NR	Sawada (2020) [15]
7	c.431G > A	p.G144D	Exon 3	NR	Hetero	Probably damaging (1.000)	+	NA	Li (2014) [38]
8	c.436C > T	p.P146S	Exon 3	Pathogenic	Mild	Probably damaging (1.000)	+	NR	Ploos van Amstel (1994) [31]
9	c.493G > A	p.D165N	Exon 3	NR	VUS?	Probably damaging (1.000)	+	NR	Sawada (2020) [15]
10	c.538 T > G	p.L180V	Exon 3	NR	VUS?	Possibly damaging (0.470)	+	NR	Sawada (2020) [15]
**11**	**c.595G > A**	**p.V199M**	**Exon 4**	**Uncertain significance**	**Classic**	**Probably damaging (1.000)**	**+**	**NR**	**Shabbeer (2002) [26]**
12	c.605G > T	p.C202F	Exon 4	NR	VUS?	Probably damaging (1.000)	−	NR	Sawada (2020) [15]
13	c.625 T > C	p.W209R	Exon 4	NR	VUS?	Benign (0.000)	NA	0.000030	Sawada (2020) [15]
14	c.628C > T	p.P210S	Exon 4	NR	Later-onset	Possibly damaging (0.758)	+	0.000015	Saito (2013) [32]
15	c.640-801G > A	−	Intron 4	Pathogenic	Later-onset	−	Unknown	NR	Ishii (2002) [19]
16	c.644 A > G	p.N215S	Exon 5	Pathogenic	Later-onset	Benign (0.048)	+	NR	Eng (1994–2) [33]
17	c.685 T > G	p.F229V	Exon 5	NR	Later-onset	Probably damaging (1.000)	−	NR	Turkmen (2016) [34]
18	c.687 T > G	p.F229L	Exon 5	NR	Variant	Possibly damaging (0.950)	+	NR	Kawano (2007) [39]
19	c.714 T > A	p.S238R	Exon 5	NR	VUS?	Probably damaging (1.000)	−	NR	Sawada (2020) [15]
20	c.719delA	p.K240Rfs*29	Exon 5	NR	Classic	−	−	NR	Lukas (2013) [27]
21	c.725 T > C	p.I242T	Exon 5	NR	Hetero	Benign (0.079)	+	NR	Tsukimura (2014) [40]
22	c.761_763delTTG	p.V254del	Exon 5	NR	Classic	−	+	NR	Sawada (2020–2) [28]
23	c.888G > A	p.M296I	Exon 6	Pathogenic	Later-onset	Benign (0.309)	+	NR	Okumiya (1995) [35]
24	c.899 T > C	p.L300P	Exon 6	Pathogenic, uncertain significance	Variant	Probably damaging (1.000)	+	NR	Shin (2007) [36]
25	c.1067G > A	p.R356Q	Exon 7	Uncertain significance, likely pathogenic	no phenotype assigned	Benign (0.190)	+	NA	Hwu (2009) [42]
**26**	**c.1072_1074delGAG**	**p.E358del**	**Exon 7**	**Pathogenic**	**Classic**	**−**	**−**	**NR**	**Blanch (1996) [17]**
27	c.1171 A > G	p.K391E	Exon 7	NR	Hetero	Benign (0.266)	+	0.000015	Wakakuri (2016) [37]
28	c.1208 T > C	p.L403S	Exon 7	NR	no phenotype assigned	Probably damaging (1.000)	+	NR	Shimotori (2008) [25]
29	c.1231G > A	p.G411S	Exon 7	NR	VUS?	Probably damaging (1.000)	+	NR	Sawada (2020) [15]

*: last updated 2021/05/10 ver.3.5, **: Galafold (migalastst) amenability table (last updated August 2021), ***: Tohoku Medical Megabank (ToMMo 38KJPN-PAR2), NR: not registered, NA: not available, VUS: variants of unknown significance.

Bold: variants receiving enzyme replacement therapy.

Table 1 lists the variants detected in 77 newborns (57 males and 20 females). The most common variant in the 77 newborns was c.436C > T (allele frequency: 9.68%), and c.640-801G > A (9.68%). The second most common variant was c.725 T > C p.I242T [40] (8.60%), followed by c.888G > A (7.53%). Thirty-five male and 14 female newborns with pathogenic variants were diagnosed as hemizygous and heterozygous, respectively. Additionally, 22 male and 6 female newborns with VUS were diagnosed as potential hemizygous and potential heterozygous patients, respectively.

### 3.3 Follow-up survey for the 77 newborns

Seventy-seven newborns underwent follow-ups and interventions by clinicians. Five hemizygous patients (No. 1: c.2 T > A, 3: c.128G > A, 19 and 20: c.595G > A, and 53: c.1072_1074delGAG) presented with signs or symptoms and underwent ERT (Table 1). Patient 1 developed extremity pain and hypohidrosis at the age of 3 years and started treatment with ERT at the age of 5. Patient 3 showed delayed myocardial enhancement on cardiac magnetic resonance imaging at the age of 5 years [41], and mulberry cells were detected in the urinary sediment, and he started treatment with ERT at the age of 6.

Patients 19 and 20 are siblings and were symptom-free. However, persistent urinary mulberry bodies and microproteinuria were detected on urinalysis, and kidney biopsies showed zebra bodies, a characteristic feature of FD. ERT was initiated at 12 and 10 years of age, respectively. Patient 53 developed extremity pain and hypohidrosis at the age of 4 years and had undergone ERT and carbamazepine treatment.

## 4 Discussion

In this study, we identified 77 newborns from 74 families with pathogenic variants or VUS using the NBS program for FD. The frequency of FD in our NBS was 1:7730 (0.013%), whereas that of pathogenic variants was 1:12,436 (0.008%) [16]. Herein, we screened an additional 182,880 newborns using Method III. The main difference between Method III and the previous methods is the number of diseases tested simultaneously, owing to the modified extraction solution (Fig. 2).

Specifically, Method I tests for FD only, Method II tests for three diseases, including FD, Pompe disease (PD), and Gaucher disease (GD), and Method III tests for FD, PD, GD, and Mucopolysaccharidosis type I and type II. However, the recall rate for Method II was 0.04%, lower than the 0.19% recall rates for Methods I and III. The difference in recall rates might have been influenced by the cutoff values used. The cutoff value for Method I was set at 50% of the median α-Gal A activity value in the general population, and the cutoff value for Method II was the same as that for Method I from the values measured using the samples measured in Method I. The cutoff value for Method III was set to the upper limit of α-Gal A activity values for individuals with pathogenic variants. Periodic checks for changes in recall and false-positive rates should be performed to establish appropriate cut-off values.

In our study population, 29 *GLA* variants were detected in 77 newborns with FD. The most common variant was c.436C > T (allele frequency: 9.68%), and c.640-801G > A (9.68%), followed by c.725 T > C (8.60%), and c.888G > A (7.53%).

The c.436C > T is classified as a pathogenic variant in ClinVar [21]. However, individuals with this variant in our study, as well as some family members, have shown no symptoms related to FD. Patients with this variant included six hemizygous males aged 0–8 years and three heterozygous females aged 0–15 years. The oldest individual identified with this variant was participant 13's 52-year-old father; there have been no clinical reports of this variant.

The c.640-801G > A variant has been reported at a remarkably high frequency in Taiwan, where approximately 1 in 875 male newborns carries this mutation, accounting for >80% of the positive cases identified by NBS [42], [43], indicating an East Asian–specific founder effect. In contrast, the c.725 T > C and c.888G > A variants have been sporadically observed in Japanese NBS programs, but have not been reported at significant frequencies in large-scale screening studies from other East Asian countries. Individuals with the c.725 T > C, including the proband's family, did not show any symptoms related to FD in our study. The probands included five heterozygous females aged 0–11 years. The oldest participant was a 75-year-old grandfather. Therefore, c.436C > T and c.725 T > C may be likely nonpathogenic or mildly pathogenic [24]. c.640-801G > A and c.888G > A variants are classified as later-onset variants [20], [35]. Patients with the c.640-801G > A were identified, especially in the NBS from Taiwan [42], [43]. c.640-801G > A and c.888G > A have been detected in several patients with late-onset hypertrophic cardiomyopathy [43], [44], [45], [46], [47], [48]. Thus, there may be undiagnosed FD patients with hypertrophic cardiomyopathy and *GLA* variants such as c.640-801G > A and c.888G > A in Japan.

A follow-up survey revealed that five patients (No. 1, 3, 19, 20, and 53) had already received ERT, three of whom (No. 1, 3, and 53) had been previously reported [16]. Their FD-related symptoms have since improved following ERT. Two siblings with c.595G > A showed no symptoms of FD. c.595G > A is a classic variant [26]. Among these patients, three with classic variants exhibited pathophysiological changes in the heart (No. 3) or kidney (No. 3, 19 and 20) before the onset of symptoms. In Japan, although ERT is generally recommended to be initiated immediately after symptom onset, we believe it should be initiated at the time FD-related signs appear. Van der Veen et al. reported that the initiation of ERT before age 16 in male patients with FD was associated with a reduced presentation rate of renal and cardiac manifestations of FD [49].

In conclusion, we performed NBS for FD in 782,591 newborns and detected 29 *GLA* variants in 77 newborns. High-frequency variants included c.640-801G > A and c.888G > A. The c.888G > A detected in our NBS study was classified as a late-onset mutation from the Fabry database classification and may cause hypertrophic cardiomyopathy in adulthood or later. Follow-up studies in our study revealed that the histological changes had already progressed before the appearance of overt clinical symptoms. Therefore, an evaluation system for clinical follow-up should be established, and the timing of ERT initiation for each individual with a pathogenic variant should be considered based on prior reports and variant information.

## Declaration of generative AI in scientific writing

ChatGPT (OpenAI, San Francisco, CA) was used for English language editing. The authors are responsible for the scientific content of this manuscript.

## CRediT authorship contribution statement

**Takaaki Sawada:** Writing – original draft. **Jun Kido:** Writing – original draft. **Keishin Sugawara:** Methodology, Data curation. **Shinichiro Yoshida:** Methodology, Data curation. **Takahito Inoue:** Investigation. **Shinichi Hirose:** Supervision, Conceptualization. **Kimitoshi Nakamura:** Project administration, Funding acquisition, Conceptualization.

## Funding

This work was supported in part by a Health and Labor Sciences Research Grant for Research on Rare and Intractable Diseases from the Ministry of Health, Labor, and Welfare, Japan (grant number: 23FC1032 and 23FC1033); the Children and Families Agency Program Grant (grant number: 23DA0801); a Grant-in-Aid for Scientific Research from the Ministry of Education, Culture, Sports, Science, and Technology, Japan (Japan Society for the Promotion of Science [JSPS] KAKENHI, grant numbers JP23K07294, JP23K07316, and JP23K14954). The funder played no role in the study design, data collection, data analysis, decision to publish, or manuscript preparation.

## Declaration of competing interest

The authors declare that they have no competing financial interests or personal relationships that may have influenced the work reported in this study.

## Data Availability

The data that support the findings of this study are available from the corresponding author upon reasonable request.
